# Supplementary figures and images for: Extracellular Vesicle-Based Method for Detecting MYCN Amplification Status of Pediatric Neuroblastoma
Source: Cancers (Basel). 2022 May 26;14(11):2627. doi: 10.3390/cancers14112627 (PMC9179557; doi:10.3390/cancers14112627)

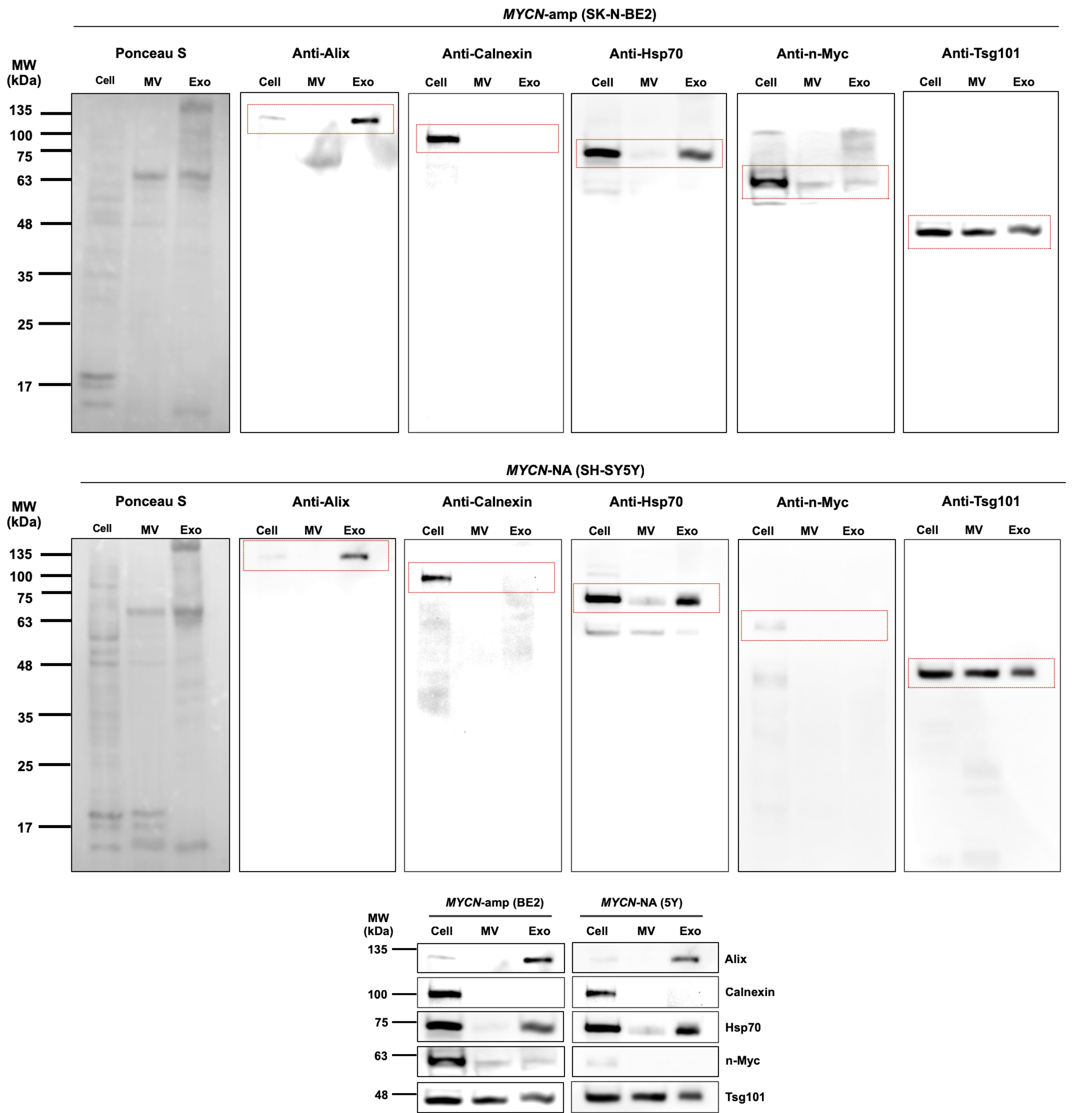

**Figure S1:** The full-length immunoblot results of the cropped images shown in Figure 1.

Supplement: Supplementary file 1 [file cancers-14-02627-s001.zip › cancers-1705896-supplementary.pdf]
